# Physical Activity Patterns Among Adolescents in the Latin American and Caribbean Region

**DOI:** 10.1123/jpah.2022-0136

**Published:** 2022-08-17

**Authors:** Antonio Bernabe-Ortiz, Rodrigo M. Carrillo-Larco

**Affiliations:** 1 CRONICAS Center of Excellence in Chronic Diseases, Universidad Peruana Cayetano Heredia, Lima, Peru; 2 Universidad Científica del Sur, Lima, Peru; 3 Department of Epidemiology and Biostatistics, School of Public Health, Imperial College London, London; 4 Universidad Continental, Lima, Peru

**Keywords:** Physical activity, physical inactivity, sedentary behavior, Latin America

## Abstract

**Background:**

Physical activity implies different patterns, but studies focused on physical inactivity and sedentary behaviors. This study aimed to estimate the prevalence of different physical activity patterns among adolescents in Latin America and the Caribbean region (LAC).

**Methods:**

Pooled analysis of the most updated data of the Global School-based Student Health surveys. Age-standardized prevalence of four outcomes was estimated using information of last 7 days: physical inactivity (0 days of at least 60min/day), insufficient physical activity (<5 days of at least 60min/day), commuting physical activity (≥5 days of walking or biking to school), and sedentary behavior (≥3 hours/day of sitting time).

**Results:**

A total of 132,071 records (33 countries) was analyzed, mean age 14.6 years, 51.2% girls. Pooled age-standardized prevalence of physical inactivity was 22.3%, greater among females (25.4%) than males (19.1%); insufficient physical activity was present in 67.7%, greater in females (73.6%) than males (61.5%); commuting physical activity was seen in 43.7%, similar between females (43.3%) and males (44.1%); and sedentary behavior was present in 43.4%, greater among females (45.4%) than males (41.3%).

**Conclusions:**

In LAC, almost two thirds of adolescents are insufficiently physically active, ≥40% are sedentary, and ≥20% are physically inactive, more frequent among girls than boys.

## Introduction

According to the World Health Organization (WHO), children and adolescents should accumulate at least 60 minutes of moderate- to vigorous-intensity physical activity per day [1]. Studies in different countries have demonstrated that physical inactivity and sedentary lifestyle behaviors are common among adolescents, especially in low- and middle-income countries (LMIC) [2, 3]. Thus, a global analysis reported that more than 80% of students between 11-17 years were insufficiently physically active in 2016 (i.e., <5 days of 60 min of moderate- to vigorous-intensity physical activity) [4], being more common among girls than boys. Such estimates were similar to those reported in an analysis of 34 mainly LMIC conducted between 2003 and 2007 [5].

Physical activity implies different patterns; however, many studies have focused on physical activity/inactivity and sedentary behaviors [6, 7] as both have been evaluated separately and been independently associated with adverse health outcomes [8-10]. Nevertheless, a deeper understanding of physical activity patterns, including commuting physical activity and insufficient physical activity, may support the implementation of appropriate prevention and intervention strategies in a specific region. This is relevant as usually physical activity declines during the transition from childhood to adolescence [11], and for instance, this period seems to be an ideal time for the adoption of physical activity behaviors [12].

Latin America and the Caribbean (LAC) region comprises LMIC countries with the highest levels of physical inactivity for both boys and girls [4]. Existing literature, using information from 2007 to 2013, shows that only 15% of adolescents in LAC countries were physically active [13]. As a result, there is a need to update and better understand the epidemiology of physical activity among adolescents in the region by using different physical activity indicators. In addition, promoting healthy physical activity patterns at home and school seems to be relevant to flatten the overnutrition epidemic. However, physical activity patterns are different by sex [6], which can have an impact on the strategies to be used to promote physical activity.

Therefore, this study aimed to describe and estimate the prevalence, overall and by sex, of different physical activity patterns (physically inactivity, insufficient physical activity, commuting physical activity, and sedentary behavior) among adolescents in the Latin American and Caribbean region. In so doing, we updated the regional evidence and expanded it by incorporating commuting physical activity.

## Methods

### Study design and setting

The Global School-based Student Health (GSHS) is a collaborative surveillance project designed to help countries measure and evaluate the behavioral risk and protective factors in 10 key areas among young subjects aged 13 to 17 years. The GSHS was developed by the United States Centers for Disease Control and Prevention (CDC), the World Health Organization (WHO) and other United Nations allies, and data for analysis is freely available [14]. For this manuscript, the most updated representative data from Latin American and the Caribbean countries were pooled for analysis.

### Sampling strategy

The GSHS used a standardized two-stage sampling approach for the selection of students within each country. In the first stage, schools were chosen with probability proportional to sample size; whereas in the second stage, a random selection of classrooms was conducted within each selected school. All students in selected classrooms were eligible to participate in the survey regardless of age [14].

The GSHS utilizes core questionnaire modules, core-expanded questions, and countryspecific questions that are combined to form a self-applied tool. Thus, the questionnaire can be administered during one regular class period [14].

The 10 core questionnaire modules address the leading causes of morbidity and mortality among children and adults globally: alcohol use, dietary behaviors, drug use, hygiene, mental health, physical activity, protective factors, sexual behaviors, tobacco use, and violence and unintentional injury [15].

### Definition of variables

Four were the outcomes of interest based on three questions of the physical activity core module. These three questions were utilized to build the outcomes as they were common across country-specific surveys.

The first outcome was physical inactivity, built based on the question “During the past 7 days, on how many days were you physically active for a total of at least 60 minutes per day?”, and the responses were based on the number of days (from 0 to 7). Thus, those who responded 0 days were categorized as physically inactive [16]. Using the same question, a second outcome, insufficient physical activity, was built using a traditional cutoff of 5 days per week. Therefore, an adolescent with <5 days of at least 60 minutes of physical activity was categorized as insufficiently active [4].

The third outcome was commuting physical activity (i.e., walking or biking to go and come from school). This outcome was built based on the question “During the past 7 days, on how many days did you walk or ride a bicycle to and from school?”, with response based on the number of days (from 0 to 7). For analysis purposes, we defined physically active as walking or biking to school for ≥5 days. This decision was done because usually school activities are carried out from Monday to Friday (i.e., 5 days per week) in the Latin America and Caribbean region [17].

Finally, the fourth outcome was sedentary behavior, based on the question “How much time do you spend during a typical or usual day sitting and watching television, playing computer games, talking with friends, or doing other sitting activities (country specific examples)?”, and possible responses were <1 hour per day, 1 to 2 hours per day, 3 to 4 hours per day, 5 to 6 hours per day, 7 to 8 hours per day, and >8 hours per day. For analysis purposes, sedentary behavior was defined as ≥3 hours of sitting time per day as in a previous report [7]. This cutoff was used as a proxy of the number of hours per day of screen-based behaviors (i.e., TV watching) [18], which has been associated with adverse health consequences [19].

Other variables included in the analysis were sex (male vs. female), age (in years), country, and survey year. Additionally, the countries were grouped into subregions within LAC using an adapted version of the NCD RisC approach [20, 21]: Andean Latin America, Caribbean, Central Latin America, and Southern Latin America (See details in Supplemental Table 1).

### Statistical analysis

All analyses were conducted in STATA 16 for Windows (StataCorp, College Station, TX, US). Prevalence and mean estimates were calculated using strata, primary sampling units and sampling weights at the country level in consideration of the complex sampling design of the GSHS. For that, we utilized the denormalized individual GSHS survey weights, considering sampling design and non-response rates.

Analyses by specific subgroups (i.e., by sex and by subregion) were done using the *subpop* command, according to literature [22]. In this latter case, the subpopulation option is used to obtain valid estimates. Thus, only the specific subgroup (i.e., females) is utilized in the estimation of the prevalence, but all participants are included in the standard errors’ estimation to obtain confidence intervals.

Using the WHO population as standard, age-standardized prevalence of the outcomes of interest were estimated, overall, by sex and by country. Differences between groups (i.e., by sex and subregion groups) were tested using the Pearson Chi-squared test with Rao-Scott correction [23]. A p-value <0.05 was considered significant.

### Ethics

Ethical approval was not sought as the present analysis used open-access surveys, and for instance datasets did not include any personal identifier.

## Results

### Overall description of the study population

GSHS surveys were conducted between 2003 (Venezuela) and 2018 (Argentina, Bolivia, Panama, St. Lucia, and St. Vincent & Grenadines). Sample sizes ranged from 212 in Montserrat (2009) to 56,981 in Argentina (2018), adding up to a total of 132,071 records in 33 countries from LAC. Data was nationally representative for 30 countries, except for Colombia, Ecuador and Venezuela as they only have subnational samples. Data available for analyses are shown in Supplemental Table 2.

A total of 7.9% of the total records had missing values in key variables for statistical analysis, varying from 2.3% in Costa Rica (2009) to 25.3% in Curacao (2015). Pooled mean age was 14.6 (SD: 1.4) years, ranging from 13.3 in Venezuela to 15.2 years in Bolivia, Dominican Republic and St. Vincent and Grenadines. The overall proportion of girls was 51.2%, varying from 47.4% in Guatemala to 56.0% in Grenada. Details are shown in Supplemental Table 3.

### Physical inactivity

Pooled age-standardized prevalence of physical inactivity was 22.3% (95% CI: 21.5% - 23.1%), but estimates ranged from 16.7% (95% CI: 14.2% - 19.5%) in Chile to 40.4% (95% CI: 35.9% - 45.2%) in Guyana. When analyses were done by subregion, the highest prevalence of physical inactivity was in the Caribbean subregion (31.7%), followed by Central Latin America (27.2%), Andean Latin America (21.7%), and finally Southern Latin America (18.0%, p<0.001).

**Figure 1:**
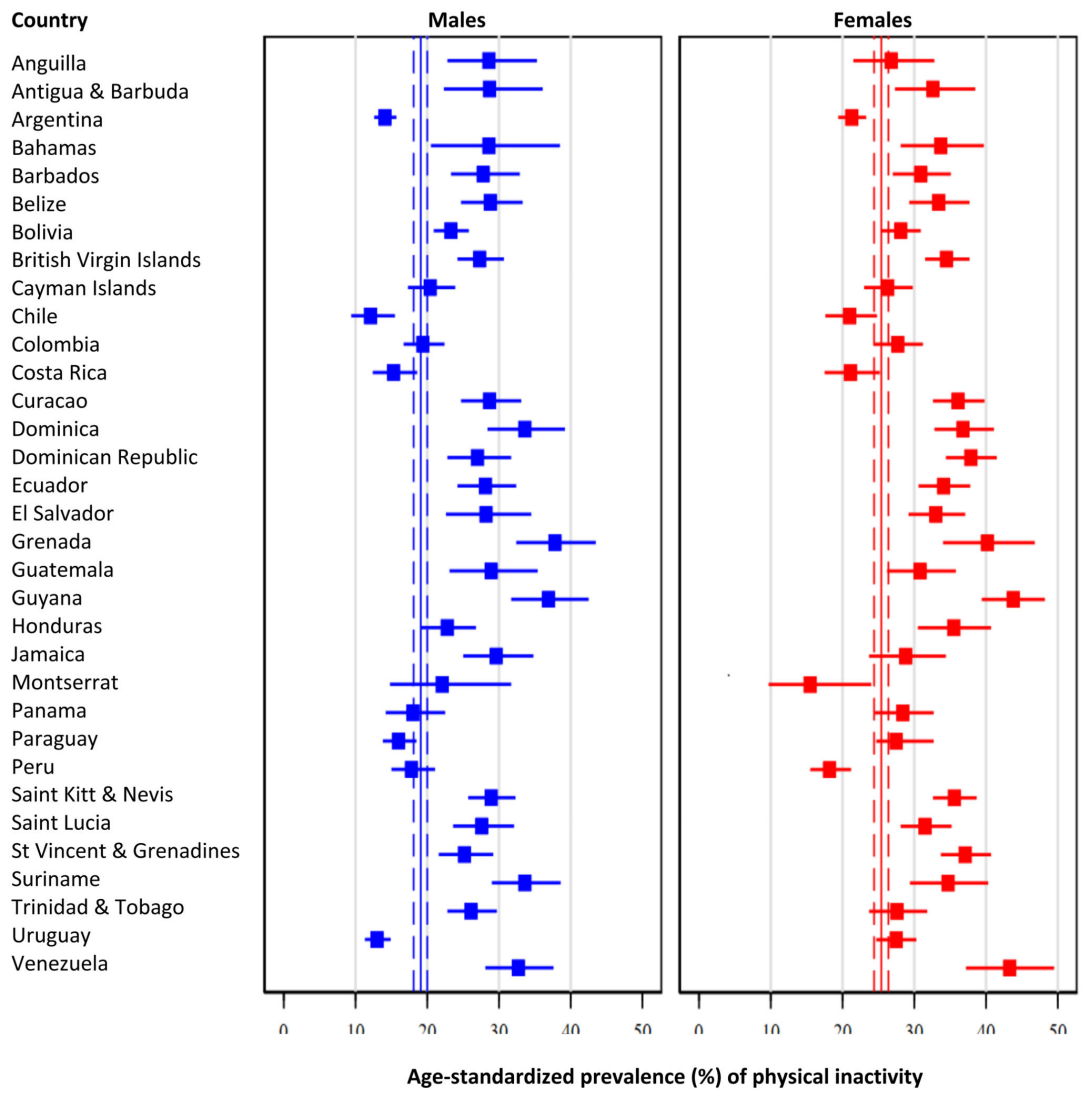
Age-standardized prevalence of physical inactivity: Results by country and sex Pooled age-standardized prevalence is shown as continuous line (point estimate) and dashed lines (95% confidence intervals).

Pooled estimates were lower among males (19.1%; 95% CI: 18.1% - 20.0%) compared to females (25.4%; 95% CI: 24.4% - 26.4%, p<0.001). See details in Figure 1 and Supplemental Table 4. Such difference was present in all the subregions: Andean Latin America (males: 20.5% vs. females: 22.9%, p=0.02), the Caribbean (males: 28.5% vs. females: 34.7%, p<0.001), in Central Latin America (males 23.8% vs. females: 30.5%, p<0.001), and Southern Latin America (males: 13.7% vs. females: 22.0%, p<0.001).

### Insufficient physical activity

Pooled age-standardized prevalence of insufficient physical activity was 67.7% (95% CI: 66.9% - 68.5%), but estimates ranged from 60.6% (95% CI: 55.4% - 65.6%) in Antigua & Barbuda to 84.4% (95% CI: 81.6% - 86.8%) in Venezuela. When analyses were done by subregion, the highest prevalence of insufficient physical activity was in the Central Latin America subregion (72.7%), followed by the Andean Latin America (70.5%), the Caribbean (69.6%), and finally Southern Latin America (63.0%, p<0.001).

**Figure 2:**
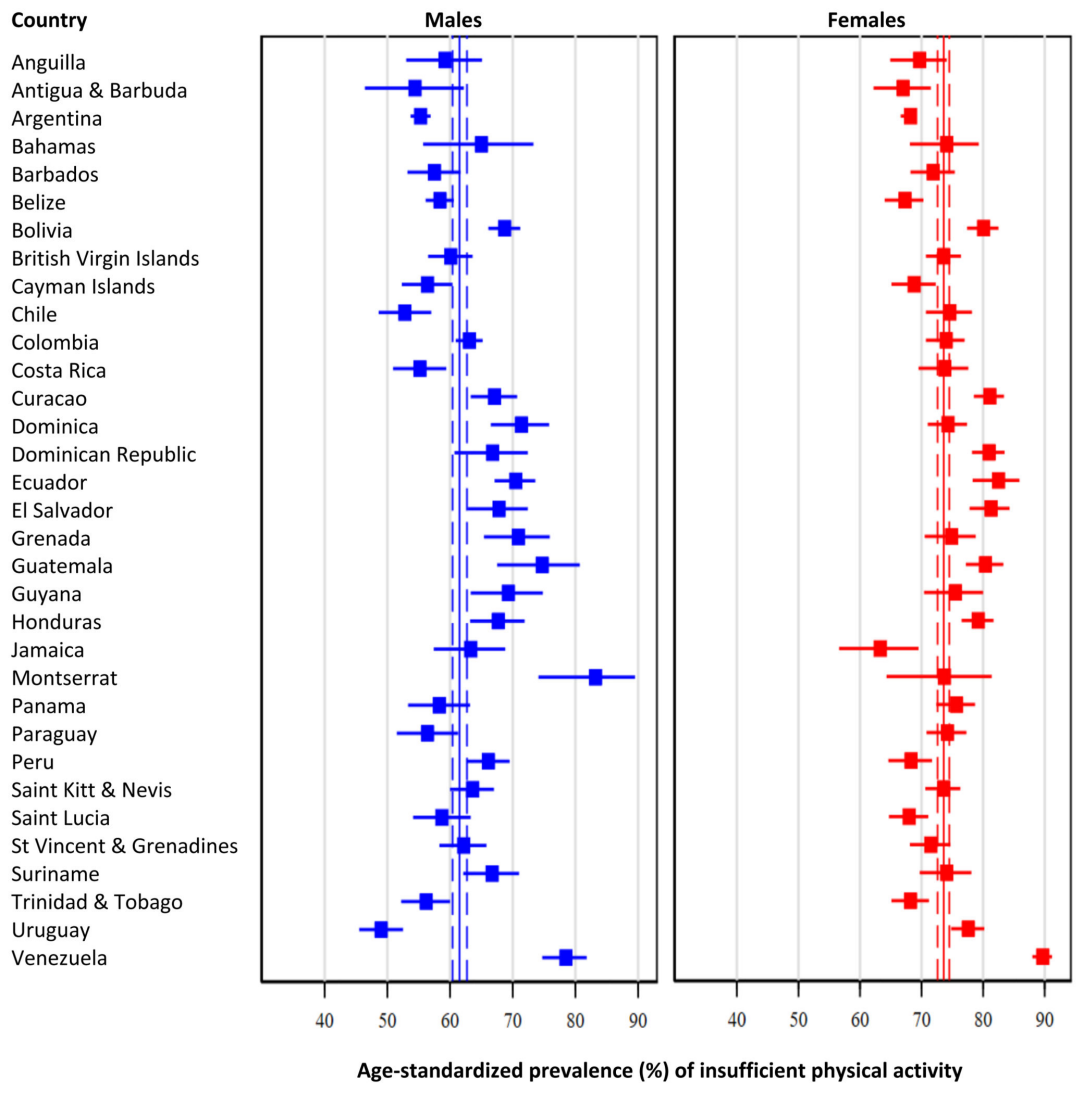
Age-standardized prevalence of insufficient physical activity: Results by country and sex Pooled age-standardized prevalence is shown as continuous line (point estimate) and dashed lines (95% confidence intervals).

Pooled estimates were lower among males (61.5%; 95% CI: 60.4% - 62.7%) compared to females (73.6%; 95% CI: 72.6% - 74.5%, p<0.001). See details in Figure 2 and Supplemental Table 5. Similarly, estimates were lower among males in all the subregions: in the Andean Latin American subregion (males: 67.4% vs. females: 73.6%, p<0.001), Caribbean (males: 64.7% vs. females: 74.2%, p<0.001), in Central Latin American (males 67.1% vs. females: 78.1%, p<0.001), and in Southern Latin American (males: 54.6% vs. females: 70.8%, p<0.001).

### Commuting physical activity

Pooled age-standardized prevalence of commuting physical activity was 43.7% (95% CI: 42.3% - 45.1%), but estimates ranged from 1.0% (95% CI: 0.2% - 3.8%) in Montserrat to 50.5% (95% CI: 45.8% - 55.2%) in Peru. In subregion analyses, the highest prevalence of commuting physical activity was seen in the Southern Latin American region (47.6%), followed by Andean Latin American (45.0%), Central Latin American (38.4%), and finally Caribbean region (36.0%, p<0.001).

**Figure 3:**
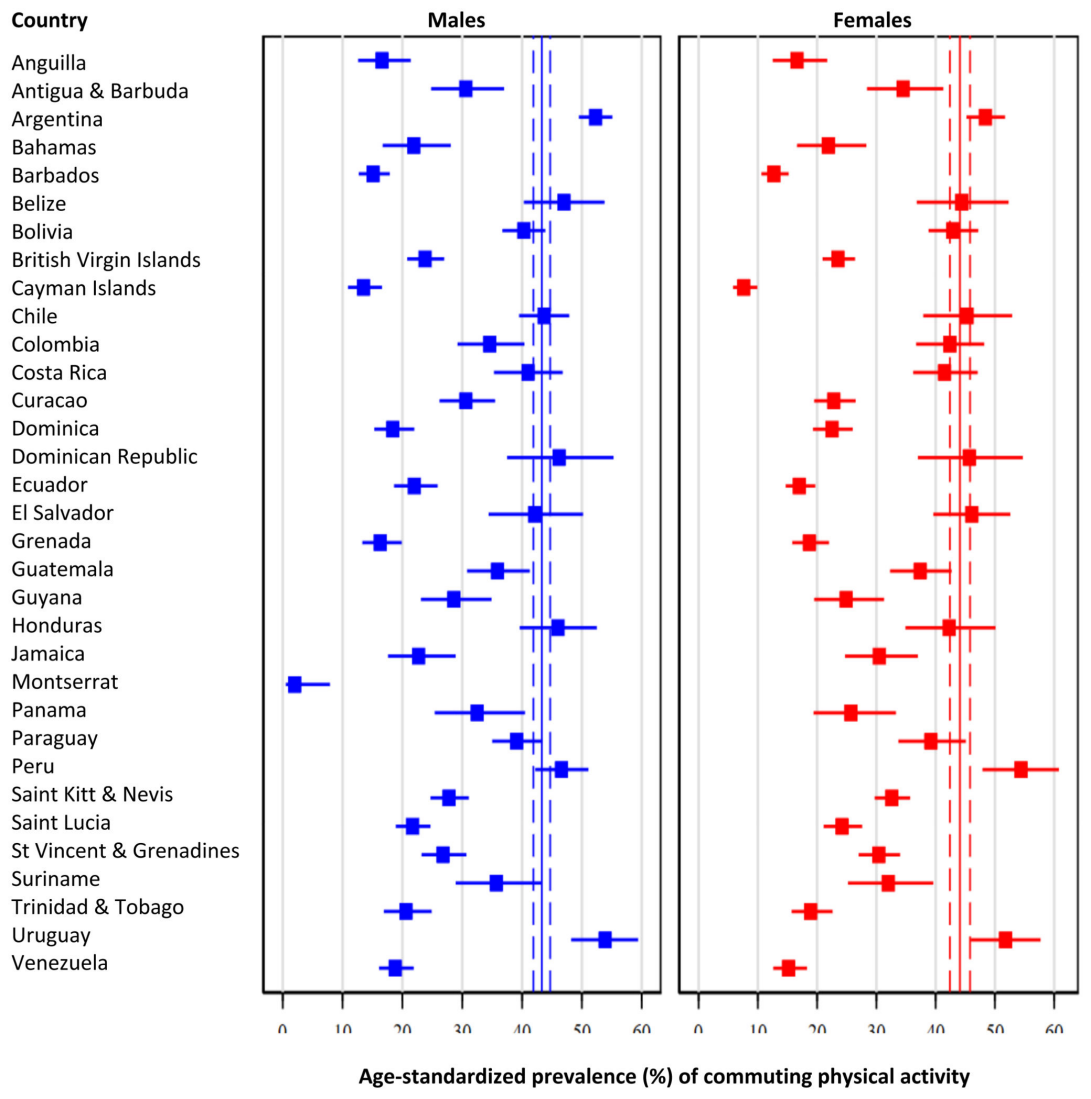
Age-standardized prevalence of commuting physical activity: Results by country and sex Pooled age-standardized prevalence is shown as continuous line (point estimate) and dashed lines (95% confidence intervals).

Pooled estimates were no different between males (43.3%; 95% CI: 41.9% - 44.7%) and females (44.1%; 95% CI: 42.4% - 45.8%, p=0.26). See details in Figure 3 and Supplemental Table 6. Estimates were no different in the Caribbean subregion (males: 35.7% vs. females: 36.3%, p=0.74), in Central Latin American (males 37.5% vs. females: 39.3%, p=0.12), and Southern Latin American (males: 48.6% vs. females: 46.7%, p=0.08); nevertheless, the difference was present in the Andean Latin American (males: 42.6% vs. females: 47.5%, p=0.004).

### Sedentary behavior

Pooled age-standardized prevalence of sedentary behavior was 43.4% (95% CI: 42.2% - 44.7%), but estimates ranged from 22.4% (95% CI: 17.2% - 28.6%) in Guatemala to 59.8% (95% CI: 57.5% - 62.1%) in St Kitts & Nevis. In subregion analyses, the highest prevalence of sedentary behavior was present in the Southern Latin American region (53.4%), followed by the Caribbean region (48.5%), Central Latin American (36.8%), and finally Andean Latin American (29.9%, p<0.001).

**Figure 4:**
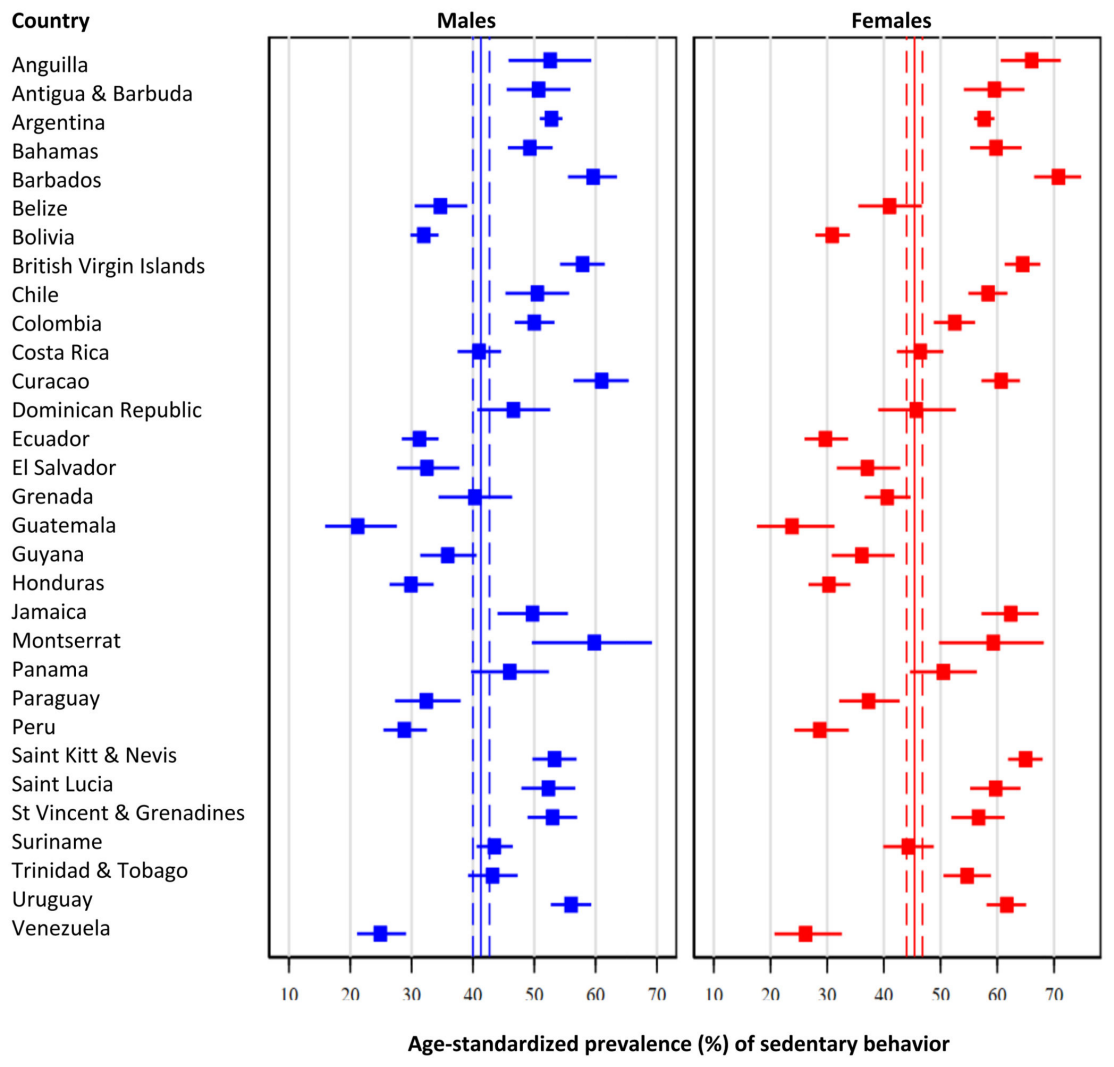
Age-standardized prevalence of sedentary behavior: Results by country and sex Pooled age-standardized prevalence is shown as continuous line (point estimate) and dashed lines (95% confidence intervals).

Pooled estimates were lower among males (41.3%; 95% CI: 40.0% - 42.7%) compared to females (45.4%; 95% CI: 44.0% - 46.8%, p<0.001). See details in Figure 4 and Supplemental Table 7. Estimates were different in the Caribbean subregion (males: 46.1% vs. females: 50.8%, p=0.03), in Central Latin American (males 34.9% vs. females: 38.7%, p=0.002), and Southern Latin American (males: 50.3% vs. females: 56.1%, p<0.001); however, the difference was not seen in the Andean Latin American (males: 30.1% vs. females: 29.7%, p=0.75).

## Discussion

### Main findings and results interpretation

Despite of the heterogeneity of physical activity patterns in the LAC region, our findings highlight that almost two thirds of adolescents are insufficiently active, more than 20% are physically inactive, less than half of participants reported commuting physical activity, and more than 40% had behaviors compatible with sedentarism. In addition, most of these unhealthy physical activity patterns are more frequent among girls than boys, and the Caribbean subregion seems to have the worst profile compared to the other subregions: the highest levels of no physical activity and the lowest level of commuting physical activity. As previously reported, such differences between countries and regions may be attributed to country income level, socioeconomic status, influence of friends, or built environment surrounding individuals [24, 25].

Our results show the alarming scenario regarding physical activity and sedentary behavior among adolescents in the LAC region, and call for large scale actions and public policies. In addition, our findings are in line with previous reports in the region [13, 26]. Adolescence is defined as a critical period of human development in which personal lifestyle elections and behavior patterns are established, including the option of being physically active [27].

As in previous studies [13, 17, 18, 26, 28], girls were less active and more sedentary than boys in almost all the countries and physical activity patterns. A study using a multilevel cross-sectional and longitudinal approach at individual, family and environmental level found that influences on physical activity at the school and family and through extracurricular sport participation are weaker among girls compared to boys [28]. Thus, different intervention approaches seem to be needed based on adolescent’s sex to guarantee appropriate levels of physical activity during this period of life.

### Relevance of results

Greater amounts of physical activity, as well as higher intensity, are associated with multiple beneficial health outcomes, including, but not limited to muscular fitness, bone health and cardiometabolic health [29]. For that reason, the WHO calls for adolescents to accumulate at least an average of 60 minute of moderate to vigorous physical activity per day (mostly aerobic physical activity) [1]. That guideline also recommends that vigorous physical activities and muscle and bone strengthening activities should each be included at least 3 days a week. Therefore, the promotion of physical activity should be mandatory to improve current and future health of adolescents, especially in countries from the Caribbean subregion, which may benefit for the implementation of multicomponent programmes at schools, but including adolescents’ perspectives in such design as girls tend to engage in different activities than boys [30]. A systematic review demonstrated that parents may play a key role and should be involved in any intervention to foster physical activity in children/adolescents [31]. In addition, multi-component strategies have been shown to be effective in increasing physical activity levels in school settings, where the adolescent spent a great proportion of their time. These multi-component strategies should include the increase of the number and quality of physical education lessons, activity breaks, after school-programmes, change in the school environment, and promotion of active transportation [32-34]. In addition, the improvement of built environment seems to be relevant [35], especially in the Caribbean and Andean Latin American subregions [36]. Traffic congestion, air pollution and traffic accidents, a great part of the population living in slums and high crime rates reduce the possibility to do physical activity. Moreover, high-quality studies on the built environment and physical activity are needed for both research and policy especially in this region [37].

On the other hand, greater time spent in sedentary behavior is also related to poorer heath outcome in adolescents [18]. Accordingly, the WHO recommends limiting the amount of time spent in sedentary behaviors among children and adolescents [1]. Some guidelines in specific countries suggest to limit recreational screen time to no more than 2 hours per day and recommend breaking up long sitting periods as often as possible [38]. Although some interventions have demonstrated to reduce sedentary behaviors among adolescents, these seem to have a small effect size [39]. Understanding the causes of sedentary behaviors, especially those related to sitting time, are relevant because this may be highly variable among countries.

### Strengths and limitations

This analysis benefits from the use of representative surveys among adolescents in different countries of the LAC region and subregions. Moreover, we analyzed more up-to-date information (up to 2018) than the most recent evidence, and advanced it by incorporating commuting physical activity. Despite of that, this study has several limitations that merit discussion. First, while GSHS follow a consistent protocol and use similar tools, the sampling procedure is not necessarily identical across countries. Although there may be different sampling procedures, the GSHS were designed to be nationally representative except in three countries (Colombia, Ecuador, and Venezuela). Readers are advised to carefully make between-country comparisons, acknowledging sampling procedures may be different and the results for three countries are not nationally representative. In addition, in post-hoc analyses, when data from these three countries was excluded, estimates slightly varied between 0.3% and 1%, data not shown). Second, the GSHS uses a self-reported approach applied at schools, and for instance, susceptible to recall and social desirability bias. In addition, questions regarding physical activity were based on the seven days prior to the application of the survey, and may not be representative of a longer life experience, raising the possibility of misclassification. Third, data on type, frequency, intensity, and duration of physical activity patterns were not collected during the application of the GSHS, and thus, metabolic equivalent could not be estimated. Fourth, sitting time was used as a proxy of sedentary behavior; however, screen use, an important behavior related to sedentarism was not evaluated as part of the survey. Fifth, as the sampling of GSHS is based on school grades, the representativeness for all the age groups included may be an issue. Finally, we utilized only the last survey data available for each of the countries involved, comprising a long period of time (2003 to 2018). In post-hoc analysis (data not shown), a reduction of physical inactivity (25.3% to 22.1%) and insufficient physical activity (70.6% to 65.9%) was seen; whereas an increment in commuting physical activity (34.4% to 44.5%) and sedentary behavior (44.7% to 47.8%) was observed. Although this approach may help to better understand the epidemiology of physical activity patterns in the region, this may be related to the high heterogeneity found in this study. Additionally, interventions to improve physical activity rates amongst children and adolescents could have been implemented since these surveys were conducted, and for instance, affect our results. This calls for a continuous surveillance of physical activity pattern among adolescents in LAC.

## Conclusions

Almost two thirds of adolescents are insufficiently physically active, ≥40% are sedentary, and ≥20% are physically inactive in LAC. These unhealthy physical activity patterns are more frequent among girls than boys, and the Caribbean subregion has the worst profile compared to the other LAC subregions.

## Supplementary Material

Supplemental Tables
