# Supplementary material for: Physical Activity Patterns Among Adolescents in the Latin American and Caribbean Region
Source: J Phys Act Health. Author manuscript; Available in PMC 2022 Sep 1. (PMC7613474; doi:10.1123/jpah.2022-0136)
Supplement: Supplemental Tables [file EMS152992-supplement-Supplemental_Tables.pdf]

SUPPLEMENTAL TABLES

Supplemental Table 1: Subregions in the Latin American and the Caribbean

**Andean Latin America (3)**	Bolivia, Ecuador, Peru
**Caribbean (19)**	Anguilla, Antigua and Barbuda, Bahamas, Barbados, Belize, British Virgin Islands, Cayman Islands, Curacao, Dominica, Dominican Republic, Grenada, Guyana, Jamaica, Montserrat, St Kitts and Nevis, St Lucia, St Vincent and the Grenadines, Suriname, Trinidad and Tobago
**Central Latin America (7)**	Colombia, Costa Rica, El Salvador, Guatemala, Honduras, Panama, Venezuela
**Southern Latin America (4)**	Argentina, Chile, Paraguay, Uruguay

Supplemental Table 2: Data available for statistical analyses

Country	Study year	Sample size	Physical activity data		
			PA time	Walking/biking	Sitting time
Anguilla	2016	813	X	X	X
Antigua & Barbuda	2009	1,266	X	X	X
Argentina	2018	56,981	X	X	X
Bahamas	2013	1,357	X	X	X
Barbados	2011	1,629	X	X	X
Belize	2011	2,112	X	X	X
Bolivia	2018	7,931	X	X	X
British Virgin Islands	2009	1,664	X	X	X
Cayman Islands	2007	1,299	X	X	X
Chile	2013	2,049	X	X	X
Colombia	2007	9,907	X	X	X
Costa Rica	2009	2,679	X	X	X
Curacao	2015	2,765	X	X	X
Dominica	2009	1,642	X	X	NA
Dominican Republic	2016	1,481	X	X	X
Ecuador	2007	5,524	X	X	X
El Salvador	2013	1,915	X	X	X
Grenada	2008	1,542	X	X	X
Guatemala	2015	4,374	X	X	X
Guyana	2010	2,392	X	X	X
Honduras	2012	1,779	X	X	X
Jamaica	2017	1,667	X	X	X
Montserrat	2009	212	X	X	X
Panama	2018	2,948	X	X	X
Paraguay	2017	3,149	X	X	X
Peru	2010	2,882	X	X	X
Saint Kitts & Nevis	2011	1,740	X	X	X
St Lucia	2018	1,970	X	X	X
St Vincent & Grenadines	2018	1,877	X	X	X
Suriname	2016	2,126	X	X	X
Trinidad & Tobago	2017	3,869	X	X	X
Uruguay	2012	3,524	X	X	X
Venezuela	2003	4,415	X	X	X

X = Data available for analysis, NA = not available; PA = Physical activity

Supplemental Table 3: Characteristics of the study population by country

Country	Missing values (%)	Age mean (SD)	Female (%)
Anguilla	7.0%	14.7 (1.3)	51.4%
Antigua & Barbuda	7.3%	14.0 (0.9)	49.0%
Argentina	5.5%	14.8 (1.3)	52.1%
Bahamas	4.9%	13.5 (1.1)	52.4%
Barbados	5.2%	14.3 (0.9)	51.2%
Belize	10.9%	14.0 (1.4)	51.8%
Bolivia	19.5%	15.2 (1.3)	49.8%
British Virgin Islands	6.6%	14.2 (1.4)	52.4%
Cayman Islands	8.2%	13.9 (1.2)	50.8%
Chile	9.9%	14.8 (1.6)	51.4%
Colombia	3.6%	14.2 (1.3)	54.7%
Costa Rica	2.3%	14.3 (1.1)	49.6%
Curacao	25.3%	14.9 (1.5)	51.4%
Dominica	9.7%	14.1 (1.4)	49.1%
Dominican Republic	14.7%	15.2 (1.3)	50.2%
Ecuador	14.4%	13.6 (1.3)	50.8%
El Salvador	4.7%	14.3 (1.1)	48.2%
Grenada	10.4%	14.1 (1.3)	56.0%
Guatemala	8.7%	14.3 (1.2)	47.4%
Guyana	5.6%	14.4 (1.0)	51.5%
Honduras	6.2%	13.9 (1.3)	52.9%
Jamaica	7.5%	15.2 (1.3)	51.9%
Montserrat	8.5%	14.3 (1.2)	53.4%
Panama	14.7%	15.1 (1.3)	53.3%
Paraguay	10.0%	14.8 (1.5)	51.4%
Peru	2.9%	14.4 (1.0)	49.6%
Saint Kitts & Nevis	4.0%	14.5 (1.0)	49.1%
St Lucia	8.4%	14.4 (1.6)	52.7%
St Vincent & Grenadines	10.4%	15.2 (1.2)	52.1%
Suriname	12.9%	14.4 (1.4)	50.9%
Trinidad & Tobago	12.4%	14.3 (1.5)	52.6%
Uruguay	3.4%	14.4 (1.0)	54.4%
Venezuela	7.6%	13.3 (1.1)	52.3%

Missing values included only key variables for analysis.

Supplemental Table 4: Age-standardized prevalence of physical inactivity: by sex and country

Country	Physical inactivity in the last 7 days		
	Total	Male	Female
Anguilla	27.7% (23.0%-32.9%)	28.6% (22.8%-35.3%)	26.8% (21.5%-32.8%)
Antigua & Barbuda	30.6% (26.1%-35.6%)	28.7% (22.3%-36.1%)	32.6% (27.3%-38.5%)
Argentina	17.8% (16.3%-19.5%)	14.1% (12.6%-15.7%)	21.3% (19.4%-23.3%)
Bahamas	31.3% (25.5%-37.7%)	28.6% (20.5%-38.5%)	33.7% (28.1%-39.7%)
Barbados	29.4% (26.6%-32.3%)	27.8% (23.3%-32.9%)	30.9% (27.0%-35.1%)
Belize	31.2% (27.5%-35.1%)	28.8% (24.7%-33.3%)	33.4% (29.3%-37.7%)
Bolivia	25.7% (23.5%-27.9%)	23.3% (20.9%-25.8%)	28.1% (25.4%-30.9%)
British Virgin Islands	31.5% (29.3%-33.8%)	27.3% (24.2%-30.7%)	34.5% (31.5%-37.7%)
Cayman Islands	23.5% (21.2%-25.9%)	20.4% (17.3%-23.9%)	26.3% (23.0%-29.8%)
Chile	16.7% (14.2%-19.5%)	12.1% (9.4%-15.5%)	21.0% (17.6%-24.8%)
Colombia	23.9% (21.6%-26.5%)	19.4% (16.7%-22.4%)	27.7% (24.4%-31.2%)
Costa Rica	18.2% (15.5%-21.2%)	15.3% (12.4%-18.6%)	21.1% (17.5%-25.2%)
Curacao	32.5% (29.6%-35.6%)	28.7% (24.7%-33.1%)	36.1% (32.6%-39.8%)
Dominica	35.2% (31.7%-38.8%)	33.6% (28.4%-39.2%)	36.8% (32.8%-41.1%)
Dominican Republic	32.5% (29.6%-35.5%)	27.0% (22.8%-31.7%)	37.9% (34.4%-41.5%)
Ecuador	31.1% (28.0%-34.4%)	28.1% (24.2%-32.4%)	34.1% (30.6%-37.8%)
El Salvador	30.5% (26.6%-34.8%)	28.2% (22.6%-34.5%)	33.0% (29.2%-37.1%)
Grenada	39.1% (34.3%-44.2%)	37.8% (32.4%-43.5%)	40.2% (34.0%-46.8%)
Guatemala	29.8% (24.9%-35.1%)	28.9% (23.1%-35.4%)	30.8% (26.2%-35.8%)
Guyana	40.4% (35.9%-45.2%)	36.9% (31.7%-42.5%)	43.8% (39.4%-48.2%)
Honduras	29.5% (25.6%-33.7%)	22.8% (19.2%-26.8%)	35.5% (30.5%-40.7%)
Jamaica	29.2% (25.2%-33.5%)	29.6% (25.0%-34.8%)	28.8% (23.7%-34.4%)
Montserrat	18.5% (13.7%-24.5%)	22.1% (14.8%-31.7%)	15.5% (9.7%-24.0%)
Panama	23.5% (20.0%-27.5%)	18.0% (14.2%-22.5%)	28.4% (24.5%-32.7%)
Paraguay	21.9% (19.8%-24.1%)	16.0% (13.8%-18.5%)	27.5% (24.7%-30.4%)
Peru	18.0% (15.5%-20.8%)	17.8% (15.0%-21.1%)	18.2% (15.5%-21.2%)
Saint Kitts & Nevis	32.7% (30.6%-35.0%)	28.9% (25.7%-32.3%)	35.6% (32.6%-38.7%)
St Lucia	29.7% (26.8%-32.7%)	27.6% (23.6%-32.1%)	31.5% (28.1%-35.2%)
St Vincent & Grenadines	31.4% (29.0%-34.0%)	25.2% (21.6%-29.2%)	37.1% (33.7%-40.7%)
Suriname	34.1% (30.2%-38.3%)	33.6% (29.0%-38.6%)	34.7% (29.4%-40.3%)
Trinidad & Tobago	26.9% (23.9%-30.1%)	26.1% (22.8%-29.7%)	27.6% (23.7%-31.8%)
Uruguay	20.9% (18.7%-23.1%)	13.0% (11.3%-14.9%)	27.5% (24.7%-30.3%)
Venezuela	38.2% (33.7%-43.0%)	32.7% (28.1%-37.6%)	43.3% (37.2%-49.5%)

Supplemental Table 5: Age-standardized prevalence of insufficient physical activity: by sex and country

Country	Physical inactivity		
	Total	Male	Female
Anguilla	64.6% (60.4%-68.6%)	59.2% (53.0%-65.1%)	69.7% (64.9%-74.1%)
Antigua & Barbuda	60.6% (55.4%-65.6%)	54.4% (46.4%-62.2%)	67.0% (62.2%-71.5%)
Argentina	62.0% (60.6%-63.4%)	55.3% (53.7%-56.9%)	68.2% (66.6%-69.7%)
Bahamas	69.8% (63.1%-75.7%)	65.0% (55.7%-73.3%)	74.1% (68.1%-79.3%)
Barbados	64.9% (62.2%-67.6%)	57.5% (53.2%-61.7%)	71.9% (68.2%-75.4%)
Belize	63.0% (60.5%-65.4%)	58.4% (56.1%-60.6%)	67.3% (64.0%-70.3%)
Bolivia	74.4% (72.1%-76.5%)	68.7% (66.1%-71.2%)	80.1% (77.4%-82.5%)
British Virgin Islands	67.8% (65.5%-70.0%)	60.1% (56.5%-63.6%)	73.6% (70.7%-76.4%)
Cayman Islands	63.0% (60.2%-65.6%)	56.4% (52.3%-60.4%)	68.8% (65.1%-72.3%)
Chile	64.0% (60.6%-67.4%)	52.8% (48.6%-57.0%)	74.6% (70.7%-78.2%)
Colombia	69.0% (67.1%-70.9%)	63.1% (60.9%-65.2%)	74.0% (70.7%-77.0%)
Costa Rica	64.4% (61.1%-67.6%)	55.2% (50.9%-59.4%)	73.8% (69.5%-77.6%)
Curacao	74.3% (72.1%-76.4%)	67.1% (63.3%-70.7%)	81.1% (78.5%-83.4%)
Dominica	72.8% (69.9%-75.6%)	71.4% (66.5%-75.8%)	74.3% (71.0%-77.4%)
Dominican Republic	73.9% (70.3%-77.3%)	66.8% (60.7%-72.4%)	81.0% (78.2%-83.5%)
Ecuador	76.6% (73.2%-79.7%)	70.5% (67.1%-73.6%)	82.5% (78.3%-85.9%)
El Salvador	74.3% (70.6%-77.7%)	67.8% (62.8%-72.4%)	81.3% (77.8%-84.3%)
Grenada	73.1% (69.7%-76.3%)	70.9% (65.4%-75.9%)	74.9% (70.5%-78.8%)
Guatemala	77.4% (72.7%-81.5%)	74.7% (67.5%-80.7%)	80.4% (77.2%-83.3%)
Guyana	72.5% (67.5%-77.1%)	69.3% (63.3%-74.8%)	75.5% (70.4%-80.0%)
Honduras	73.8% (71.1%-76.3%)	67.7% (63.2%-71.9%)	79.2% (76.5%-81.7%)
Jamaica	63.3% (58.2%-68.1%)	63.3% (57.4%-68.8%)	63.3% (56.6%-69.5%)
Montserrat	78.5% (72.2%-83.7%)	83.2% (74.1%-89.5%)	73.7% (64.3%-81.4%)
Panama	67.6% (63.8%-71.2%)	58.3% (53.3%-63.2%)	75.7% (72.4%-78.7%)
Paraguay	65.6% (62.0%-68.9%)	56.4% (51.5%-61.3%)	74.2% (70.8%-77.3%)
Peru	67.2% (64.3%-70.0%)	66.1% (62.6%-69.5%)	68.3% (64.6%-71.7%)
Saint Kitts & Nevis	69.1% (66.8%-71.2%)	63.6% (60.0%-67.0%)	73.6% (70.6%-76.3%)
St Lucia	63.6% (60.5%-66.6%)	58.7% (54.1%-63.3%)	68.0% (64.7%-71.1%)
St Vincent & Grenadines	67.0% (64.6%-69.4%)	62.2% (58.3%-65.8%)	71.5% (68.1%-74.7%)
Suriname	70.5% (66.6%-74.0%)	66.7% (62.1%-71.0%)	74.1% (69.7%-78.1%)
Trinidad & Tobago	62.5% (59.4%-65.5%)	56.2% (52.2%-60.0%)	68.2% (65.1%-71.2%)
Uruguay	64.5% (61.5%-67.5%)	49.0% (45.5%-52.5%)	77.6% (74.8%-80.2%)
Venezuela	84.4% (81.6%-86.8%)	78.5% (74.7%-81.8%)	89.7% (88.0%-91.2%)

Supplemental Table 6: Age-standardized prevalence of commuting physical activity by sex and country

Country	Commuting physical activity		
	Total	Male	Female
Anguilla	16.6% (13.7%-19.9%)	16.6% (12.6%-21.4%)	16.6% (12.5%-21.7%)
Antigua & Barbuda	32.5% (27.0%-38.6%)	30.6% (24.8%-37.0%)	34.5% (28.4%-41.3%)
Argentina	50.3% (47.3%-53.2%)	52.3% (49.5%-55.1%)	48.4% (45.2%-51.7%)
Bahamas	21.9% (17.2%-27.4%)	21.9% (16.7%-28.1%)	21.9% (16.6%-28.3%)
Barbados	13.9% (12.1%-15.8%)	15.1% (12.7%-17.9%)	12.7% (10.6%-15.2%)
Belize	45.6% (38.6%-52.8%)	47.0% (40.3%-53.8%)	44.4% (36.8%-52.3%)
Bolivia	41.6% (38.1%-45.2%)	40.3% (36.7%-43.9%)	42.9% (38.8%-47.2%)
British Virgin Islands	23.8% (21.8%-25.9%)	23.8% (20.8%-27.0%)	23.5% (20.9%-26.4%)
Cayman Islands	10.5% (8.9%-12.3%)	13.5% (10.9%-16.6%)	7.6% (5.8%-9.9%)
Chile	44.5% (39.2%-49.9%)	43.7% (39.5%-47.9%)	45.3% (37.9%-52.9%)
Colombia	61.2% (55.7%-66.4%)	34.6% (29.2%-40.4%)	42.4% (36.7%-48.2%)
Costa Rica	41.2% (36.2%-46.4%)	41.0% (35.3%-46.8%)	41.5% (36.2%-47.1%)
Curacao	26.6% (23.3%-30.2%)	30.6% (26.2%-35.5%)	22.8% (19.5%-26.5%)
Dominica	20.4% (18.0%-23.1%)	18.4% (15.3%-22.0%)	22.5% (19.3%-26.0%)
Dominican Republic	46.0% (38.0%-54.2%)	46.2% (37.5%-55.3%)	45.7% (37.0%-54.7%)
Ecuador	19.5% (16.8%-22.4%)	22.0% (18.6%-25.9%)	17.0% (14.7%-19.7%)
El Salvador	44.0% (38.0%-50.3%)	42.1% (34.4%-50.2%)	46.1% (39.6%-52.6%)
Grenada	17.7% (15.5%-20.1%)	16.3% (13.3%-19.9%)	18.7% (15.8%-22.0%)
Guatemala	36.6% (32.0%-41.4%)	35.9% (30.8%-41.3%)	37.4% (32.3%-42.7%)
Guyana	26.7% (21.7%-32.4%)	28.6% (23.1%-34.9%)	24.9% (19.5%-31.3%)
Honduras	44.0% (38.0%-50.2%)	46.0% (39.6%-52.5%)	42.3% (34.9%-50.1%)
Jamaica	26.8% (22.0%-32.2%)	22.7% (17.6%-28.9%)	30.5% (24.7%-37.0%)
Montserrat	1.0% (0.2%-3.8%)	2.0% (0.5%-7.9%)	---
Panama	28.9% (22.5%-36.2%)	32.5% (25.4%-40.5%)	25.7% (19.4%-33.3%)
Paraguay	39.2% (34.7%-43.8%)	39.1% (35.0%-43.4%)	39.2% (33.7%-45.1%)
Peru	50.5% (45.8%-55.2%)	46.6% (42.2%-51.1%)	54.4% (47.9%-60.8%)
Saint Kitts & Nevis	30.5% (28.3%-32.7%)	27.8% (24.7%-31.1%)	32.6% (29.7%-35.7%)
St Lucia	23.0% (20.9%-25.3%)	21.7% (18.9%-24.7%)	24.2% (21.1%-27.6%)
St Vincent & Grenadines	28.7% (26.1%-31.5%)	26.8% (23.2%-30.7%)	30.4% (27.0%-34.0%)
Suriname	33.8% (27.5%-40.7%)	35.7% (28.9%-43.2%)	32.0% (25.2%-39.6%)
Trinidad & Tobago	19.7% (17.2%-22.5%)	20.6% (16.9%-24.9%)	18.9% (15.7%-22.6%)
Uruguay	52.7% (47.4%-58.0%)	53.9% (48.2%-59.4%)	51.8% (45.9%-57.7%)
Venezuela	16.9% (14.7%-19.5%)	18.8% (16.1%-21.9%)	15.2% (12.6%-18.3%)

Supplemental Table 7: Age-standardized prevalence of sedentary behavior by sex and country

Country	Sedentary behavior		
	Total	Male	Female
Anguilla	59.5% (54.1%-64.7%)	52.6% (45.8%-59.3%)	66.1% (60.6%-71.2%)
Antigua & Barbuda	55.0% (50.7%-59.1%)	50.7% (45.5%-55.9%)	59.5% (54.1%-64.8%)
Argentina	55.3% (53.6%-57.1%)	52.8% (50.9%-54.6%)	57.7% (55.9%-59.5%)
Bahamas	54.9% (51.5%-58.2%)	49.3% (45.7%-53.0%)	59.8% (55.2%-64.3%)
Barbados	65.3% (62.1%-68.4%)	59.6% (55.5%-63.5%)	70.8% (66.5%-74.8%)
Belize	38.0% (33.9%-42.2%)	34.7% (30.5%-39.1%)	41.0% (35.5%-46.7%)
Bolivia	31.5% (29.1%-33.9%)	32.0% (29.8%-34.4%)	30.9% (27.9%-34.0%)
British Virgin Islands	61.6% (59.2%-63.9%)	57.9% (54.2%-61.5%)	64.5% (61.3%-67.6%)
Chile	54.6% (51.1%-58.0%)	50.5% (45.3%-55.7%)	58.4% (54.9%-61.8%)
Colombia	51.4% (48.5%-54.2%)	50.0% (46.8%-53.3%)	52.5% (48.8%-56.1%)
Costa Rica	43.7% (40.3%-47.1%)	41.0% (37.5%-44.6%)	46.4% (42.3%-50.5%)
Curacao	60.8% (58.0%-63.6%)	61.0% (56.4%-65.4%)	60.7% (57.2%-64.0%)
Dominican Republic	46.2% (41.5%-50.9%)	46.6% (40.7%-52.6%)	45.7% (39.0%-52.7%)
Ecuador	30.5% (27.7%-33.4%)	31.3% (28.4%-34.4%)	29.7% (26.0%-33.7%)
El Salvador	34.7% (30.2%-39.5%)	32.5% (27.6%-37.8%)	37.1% (31.7%-42.9%)
Grenada	40.5% (37.1%-43.9%)	40.3% (34.4%-46.4%)	40.6% (36.6%-44.7%)
Guatemala	22.4% (17.2%-28.6%)	21.2% (15.9%-27.6%)	23.8% (17.6%-31.3%)
Guyana	36.0% (31.5%-40.8%)	35.9% (31.4%-40.6%)	36.1% (30.8%-41.9%)
Honduras	30.1% (27.5%-32.8%)	29.9% (26.4%-33.6%)	30.3% (26.7%-34.1%)
Jamaica	56.3% (51.4%-61.2%)	49.7% (44.0%-55.5%)	62.4% (57.2%-67.3%)
Montserrat	59.2% (52.3%-65.8%)	59.8% (49.6%-69.2%)	59.3% (49.7%-68.2%)
Panama	48.4% (42.6%-54.2%)	46.0% (39.7%-52.4%)	50.5% (44.6%-56.4%)
Paraguay	34.9% (30.1%-40.0%)	32.4% (27.3%-38.0%)	37.3% (32.1%-42.8%)
Peru	28.8% (25.5%-32.3%)	28.8% (25.4%-32.5%)	28.7% (24.2%-33.8%)
Saint Kitts & Nevis	59.8% (57.5%-62.1%)	53.3% (49.7%-56.9%)	65.0% (61.9%-68.0%)
St Lucia	56.2% (52.8%-59.6%)	52.3% (47.9%-56.7%)	59.7% (55.2%-64.1%)
St Vincent & Grenadines	54.9% (51.6%-58.1%)	53.0% (48.9%-57.0%)	56.7% (51.9%-61.3%)
Suriname	43.9% (40.7%-47.2%)	43.5% (40.6%-46.5%)	44.3% (39.9%-48.8%)
Trinidad & Tobago	49.3% (46.0%-52.6%)	43.2% (39.2%-47.3%)	54.7% (50.5%-58.9%)
Uruguay	59.1% (56.3%-61.8%)	56.0% (52.7%-59.3%)	61.7% (58.1%-65.1%)
Venezuela	25.6% (22.8%-28.6%)	24.9% (21.1%-29.1%)	26.2% (20.7%-32.6%)
